# Dataset of 130 metagenome-assembled genomes of healthy and diseased broiler chicken caeca from Pakistan

**DOI:** 10.1016/j.dib.2024.110487

**Published:** 2024-05-01

**Authors:** Aqsa Ameer, Farrukh Saleem, Ciara Keating, Ozan Gundogdu, Umer Zeeshan Ijaz, Sundus Javed

**Affiliations:** a Department of Biosciences, COMSATS University Islamabad, Pakistan; b Water & Environment Research Group, University of Glasgow, Mazumdar-Shaw Advanced Research Centre, Glasgow, UK; c Department of Engineering, Durham University, Durham, DH1 3LE, UK; d School of Biodiversity, One Health, and Veterinary Medicine, College of Medical, Veterinary and Life Sciences, University of Glasgow, Glasgow, UK; e Department of Infection Biology, Faculty of Infectious and Tropical Diseases, London School of Hygiene and Tropical Medicine, London, UK; f Department of Molecular and Clinical Cancer Medicine, University of Liverpool, Liverpool, UK; g College of Science and Engineering, University of Galway, Ireland

## Abstract

This article presents metagenomic-assembled genomes (MAGs) of prokaryotic organisms originating from chicken caeca. The samples originate from broiler chickens, one group was infected with Newcastle Disease Virus (NDV) and one uninfected control group. There were four birds per group. Both groups were raised on commercially available antibiotic free feed under a semi-controlled setup. The binning step of the samples identified 130 MAGs with ≥50 % completion, and ≤10 % contamination. The data presented includes sequences in FASTA format, tables of functional annotation of genes, and data from two different approaches for phylogenetic tree construction using these MAGs. Major geochemical cycles at community level including carbon, sulfur, and nitrogen cycles are also presented.

Specifications Table

**Table:** 

Subject	Biological Sciences: Microbiology: Microbiome
Specific subject area	Caecal microbial communities of broilers
Type of data	FASTA files/Tables
How the data was acquired	Illumina TruSeq ensuring ∼20M reads per samples for 8 samples using 2 × 100bp reads
Data format	Raw and Analysed
Description of data collection	The genomic DNA was extracted from the caecal samples collected from healthy (n=4) and diseased broilers (challenged with Newcastle disease virus; n=4). Genomes of 130 prokaryotic species (both bacteria and archaea) were reconstructed from the metagenome datasets
Data source location	City/Country: Islamabad/Pakistan; Latitude and Longitude: 33.6844° N, 73.0479° E
Data accessibility	Figshare: http://dx.doi.org/10.6084/m9.figshare.24901878

## 1 Value of the Data

• This data provides information about bacterial and archaeal genomes in caecum of both healthy and NDV infected broilers chickens.
• The functional potential of genomes will be beneficial for developing intervention strategies that modulate microbiome.
• Data is applicable for comparative genomic study of 130 different candidates of prokaryotes.
• Data will help to expand the knowledge of microbe-microbe and host-microbe interaction.

## 2 Data Description

**Fig. 1:**
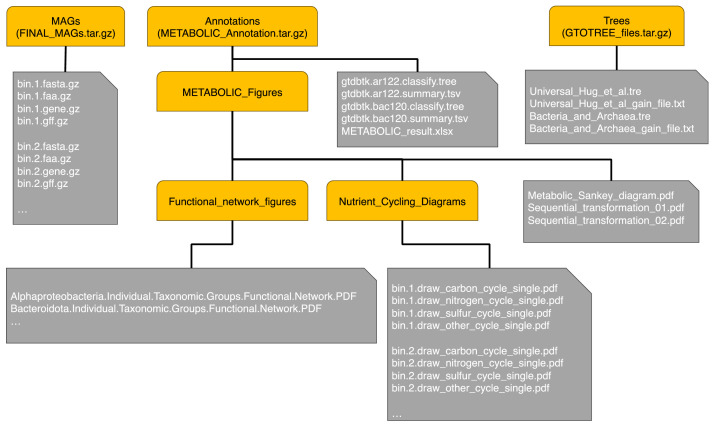
Repository structure diagram. There are three archives that provide the sequencing data and annotation for the MAGs. The yellow rounded corner nodes represent directories or compressed directories, whilst the grey node represent files. The ellipses represent a repeat of such files for each MAG.

The structure of the repository is shown in Fig. 1, where for a total of 130 metagenome-assembled genomes (MAGs), the following files are provided (x replaces the MAG number) in the FINAL_MAGs main directory:

• bin.x.fasta.gz: recovered genomic sequence
• bin.x.gene.gz: recovered genomic sequences for genes
• bin.x.faa.gz: recovered protein sequences for genes
• bin.x.gff.gz: detailed annotation of MAGs including different types of features and their locations

The METABOLIC_result.xlsx in the METABOLIC_Annotations main directory is a spreadsheet that contains 6 sheets:

• “HMMHitNum”: Presence or absence of custom HMM profiles within each MAG, the number of times the HMM profile was identified within a MAG, and the ORF(s) that represent the identified protein.
• “FunctionHit”: Presence or absence of sets of proteins which were identified and displayed as separate proteins in the sheet titled “HMMHitNum”. For each MAG, the functions are identified as “Present” or “Absent”.
• “KEGGModuleHit”: Annotation of each MAG with modules from the KEGG database organized by metabolic category. For each MAG, the modules are identified as “Present” or “Absent”.
• “KEGGModuleStepHit”: Presence or absence of modules from the KEGG database within each MAG separated into the steps that make up the module. For each MAG, the module steps are identified as “Present” or “Absent”.
• “dbCAN2Hit”: The dbCAN2 annotation results against all MAGs (CAZy numbers and hits). For each MAG, there are two distinct columns, which show the number of times a CAZy was identified and what ORF(s) represent the protein.
• “MEROPSHit”: The MEROPS peptidase searching result (MEROPS peptidase numbers and hits). For each MAG, there are two distinct columns, which show the number of times a peptidase was identified and what ORF(s) represent the protein.

The GTDB-Tk files in the METABOLIC_Annotations main directory are provided as:

• gtdtbtk.ar122.classify.tree: Tree in newick format for MAGs classified as archaea
• gtdtbtk.ar122.summary.tsv: Taxonomic classification of MAGs classified archaea at different ranks
• gtdtbtk.bac120.classify.tree: Tree in newick format for MAGs classified as bacteria
• gtdtbtk.bac120.summary.tsv: Taxonomic classification of MAGs classified as bacteria at different ranks

The Nutrient_Cycling_Diagrams directory is the sub-directory of METABOLIC_Figures of the METABOLIC_Annotations main directory. It contains the following files for each MAG (x replaces the MAG number) where in the diagrams a red arrow designates presence of a pathway step and a black arrow means absence:

• bin.x.draw_sulfur_cycle_single.PDF
• bin.x.draw_nitrogen_cycle_single.PDF .
• bin.x.draw_other_cycle_single.PDF
• bin.x.draw_carbon_cycle_single.PDF

In addition, the folder contains the summary diagrams for pathways at a community scale:

• draw_sulfur_cycle_total.PDF
• draw_other_cycle_total.PDF
• draw_nitrogen_cycle_total.PDF
• draw_carbon_cycle_total.PDF

Two sequential transformation diagrams Sequential_transformation_01.pdf and Sequential_transformation_02.pdf are provided which summarize and visualise the MAG numbers and coverages that were putatively involved in the sequential transformation of both important inorganic elements and organic compounds. Metabolic_Sankey_diagram.pdf shows the function fractions that are contributed by various microbial groups in a given community.

The Functional_network_figures directory is the sub-directory of METABOLIC_Figures of the METABOLIC_Annotations main directory. It contains diagrams representing metabolic connections of biogeochemical cycling steps at both phylum level and the whole community level.

The GTOTREE_files main directory contains the following files:

• Universal_Hug_et_al.tre: Phylogenetic tree for MAGs in Newick format recovered using 16 gene SCGs.
• Universal_Hug_et_al_gain_file.txt: Phylogenetic gain (absolute and in percentages) calculated for each MAG against all other MAGs, serving as a means to ascertain novelty.
• Bacteria_and_Archaea.tre: Phylogenetic tree for MAGs in Newick format recovered using 25 gene SCGs.
• Bacteria_and_Archaea_gain_file.txt: Phylogenetic gain (absolute and in percentages) calculated for each MAG against all other MAGs.

## 3 Experimental Design, Materials and Methods

Metagenomic microbial analysis was carried out on the caeca of from broiler chickens, including one group challenged with Newcastle disease virus and one uninfected healthy control group (4 samples per group). Both groups were raised on commercially available antibiotic-free feed under a semi-controlled setup. The experiment was conducted for 8 weeks. Challenged birds were orally challenged with Newcastle disease virus (Strain ID: chicken/broiler-25days/Sargodha/2020/PRI provided by Poultry Research Institute, Rawalpindi, Pakistan) at 6th week according to the calculated dose i.e. Embryo infectious dose_50_ (EID_50_) and caecal samples were collected from the diseased birds on 3rd day post-challenge. Birds were euthanized and caecal samples were collected aseptically and maintained at -80°C. DNA was extracted using extraction kit (Invitrogen PureLink™ Microbiome DNA Purification Kit), followed by quality check through NanoDrop spectrophotometer. Shotgun sequencing was performed on Illumina TruSeq ensuring ∼20M reads per samples for 8 samples using 2 × 100bp reads at Glasgow Polyomics sequencing facility.

**Fig. 2:**
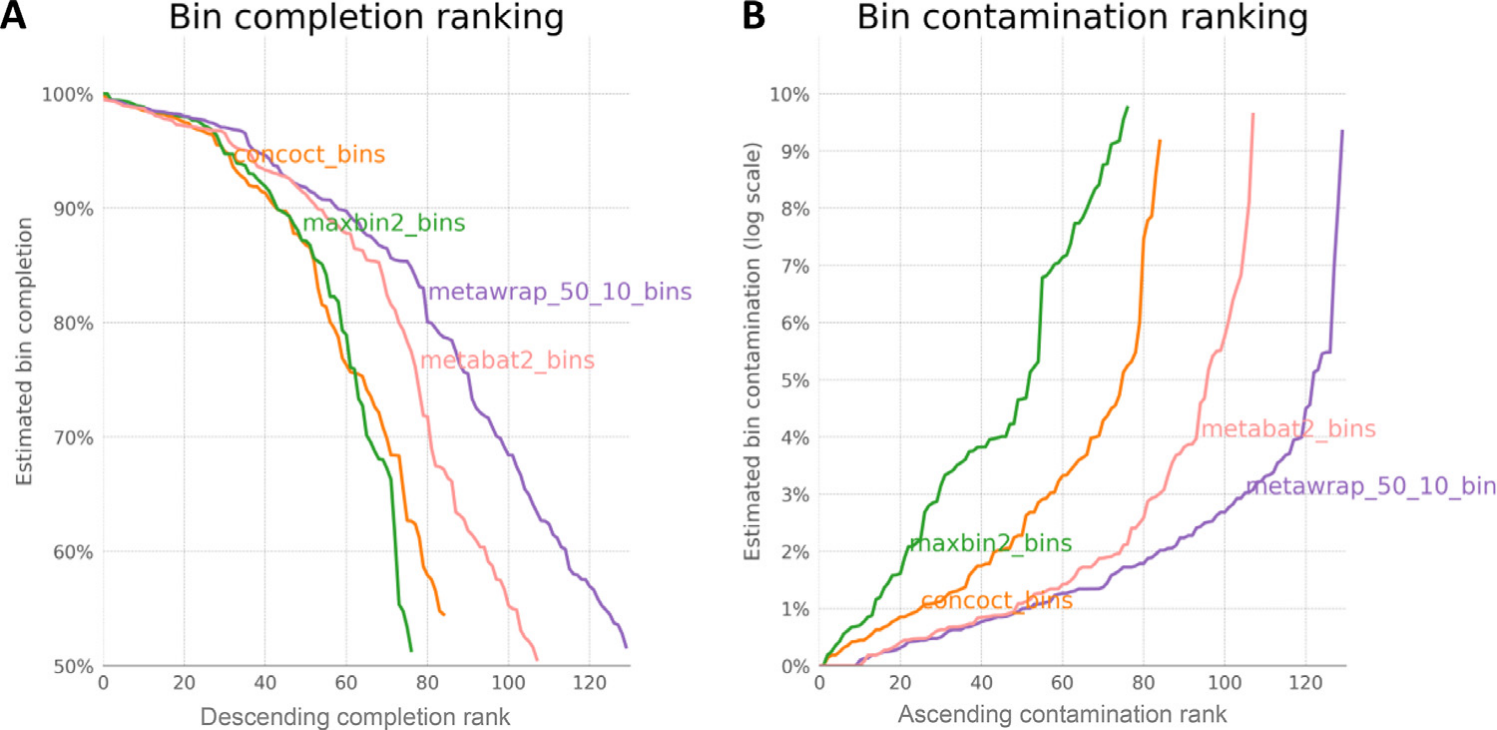
Completion (A) and contamination (B) statistics of bins recovered using original software (metaBAT2, MaxBin2, and CONCOCT) which were later refined by MetaWRAP using ≥50 % completion, and ≤10 % contamination criteria to obtain a final set of 130 MAGs.

Adapter trimmed reads were provided by the sequencing facility. These reads were then further trimmed using Sickle v1.200 [1] by trimming reads where the average Phred quality dropped below 20 and retaining paired-end reads after trimming if the length of the reads is greater than 50bp. This gave us a total of 163,357,856 quality-trimmed reads from all samples. We then collated all the forward and reverse reads together, and did an all samples co-assembly using megahit with the parameters –k-list 27,47,67,87 –kmin-1pass -m 0.95 –min-contig-len 1000 [2]. This resulted in a total of 202,665 contigs, a total of 1,396,298,223 base pairs (bp), maximum of 344,753 bp, average length of 6,890 bp and an N50 score of 16,582 bp. We then used MetaWRAP pipeline [3] and binned the contigs using three different binning algorithms: metabat2 (198 bins) [4], maxbin2 (167 bins) [5], and CONCOCT (253 bins) [6]. On these bins, we applied CheckM [7] to assess their completion as well as contamination, and within MetaWRAP framework, the bins from the three binners were consolidated together only retaining bins with ≥50 % completion, and ≤10 % contamination to give a final set of 130 bins or metagenomic assembled genomes (MAGs), with the completion and contamination ranking given in Fig. 2. For the bins, we obtained a mean genome completion of 82.90 % and a mean contamination of 1.79 %. The summary statistics of these MAGs are given in Table 1.

**Table 1:** Summary statistics of MAGs recovered using MetaWRAP pipeline with the ≥50 % completion, and ≤10 % contamination criteria based on CheckM software. The GTDB-TK classification is also shown along with the percentage gain (PG) scores for each MAG (whether included in the resulting tree) using the 25 genes Bacteria and Archaea SCGs, and the 16 genes SCGs set from [17]. The highlighted bins represent the MAGs on which either of the SCG sets worked successfully to make them part of the phylogenetic trees.

Bin	No of contigs	Average length of contigs	Total length	N50 score	Average GC %	Completeness %	Contamination %	CheckM Lineage	GTDBTK Classification	PG (Bacterial and Archaeal SCGs)	PG % (Bacterial and Archaeal SCGs)	PG (Universal Hug et al. 2016 SCGs) [17]	PG % (Universal Hug et al. 2016 SCGs) [17]
bin.1	91	33829.3	3078470	58053	46.9806	97.07	0.279	Bacteroidales	d__Bacteria;p__Bacteroidota;c__Bacteroidia;o__Bacteroidales;f__Bacteroidaceae;g__Phocaeicola;s__Phocaeicola barnesiae				
bin.10	21	87273.9	1832752	119404	55.4559	99.51	0.48	Bacteroidetes	d__Bacteria;p__Bacteroidota;c__Bacteroidia;o__Bacteroidales;f__Rikenellaceae;g__Alistipes;s__Alistipes sp900550925	0.095316	0.423142	0.061596	0.377378
bin.100	419	2580.88	1081389	2663	36.3752	63.41	0.22	Bacteria	d__Bacteria;p__Cyanobacteria;c__Vampirovibrionia;o__Gastranaerophilales;f__RUG14156;g__;s__	0.094378	0.41898	0.069035	0.422958
bin.101	215	10424	2241170	14866	63.7531	96.97	3.541	Bacteroidetes	d__Bacteria;p__Bacteroidota;c__Bacteroidia;o__Bacteroidales;f__Rikenellaceae;g__Alistipes;s__Alistipes sp900290115				
bin.102	563	4047.97	2279005	4674	52.1725	77.45	0.881	Lachnospiraceae	d__Bacteria;p__Firmicutes_A;c__Clostridia;o__Lachnospirales;f__Lachnospiraceae;g__Eubacterium_I;s__	0.122742	0.544899	0.062302	0.381703
bin.103	329	10321.9	3395913	18946	60.7765	88.76	3.076	Clostridiales	d__Bacteria;p__Firmicutes_A;c__Clostridia;o__Oscillospirales;f__Oscillospiraceae;g__Flavonifractor;s__				
bin.105	400	3439.39	1375756	3697	66.57	53.73	1.785	Bacteria	d__Bacteria;p__Firmicutes_A;c__Clostridia;o__Oscillospirales;f__Ruminococcaceae;g__Faecalibacterium;s__				
bin.106	853	1911.54	1630546	1973	59.33	68.41	2.016	Clostridia	d__Bacteria;p__Firmicutes_A;c__Clostridia_A;o__Christensenellales;f__CAG-74;g__JAAYOH01;s__	0.1075	0.477234		
bin.107	105	17238.2	1810011	23966	32.2468	98.69	0.261	Lactobacillales	d__Bacteria;p__Firmicutes;c__Bacilli;o__Lactobacillales;f__Lactobacillaceae;g__Ligilactobacillus;s__Ligilactobacillus salivarius	0.127907	0.567826	0.076622	0.469438
bin.108	343	2625.4	900512	2695	46.8817	56.64	2.495	Selenomonadales	d__Bacteria;p__Firmicutes_C;c__Negativicutes;o__Acidaminococcales;f__Acidaminococcaceae;g__Phascolarctobacterium;s__Phascolarctobacterium sp000436095	0.323812	1.437522	0.267877	1.641197
bin.109	314	11180.2	3510570	18428	44.8907	96.55	0.449	Bacteroidales	d__Bacteria;p__Bacteroidota;c__Bacteroidia;o__Bacteroidales;f__Bacteroidaceae;g__Phocaeicola;s__Phocaeicola sp002161565				
bin.11	443	4458.8	1975250	5662	59.3806	94.3	0.61	Clostridia	d__Bacteria;p__Firmicutes_A;c__Clostridia_A;o__Christensenellales;f__CAG-138;g__UBA5394;s__UBA5394 sp003150565	0.236575	1.050244	0.196704	1.205142
bin.110	235	7553.71	1775121	12513	36.4392	95.12	0.432	Lactobacillus	d__Bacteria;p__Firmicutes;c__Bacilli;o__Lactobacillales;f__Lactobacillaceae;g__Lactobacillus;s__Lactobacillus gallinarum	0.074339	0.330018	0.077128	0.472539
bin.111	143	19394.8	2773457	28886	60.5169	97.27	0.248	Clostridiales	d__Bacteria;p__Firmicutes_A;c__Clostridia;o__Oscillospirales;f__Ruminococcaceae;g__Fournierella;s__Fournierella sp002161595	0.113387	0.503364	0.073857	0.452497
bin.112	1045	2002.65	2092772	2120	61.083	62.65	2.016	Deltaproteobacteria	d__Bacteria;p__Desulfobacterota;c__Desulfovibrionia;o__Desulfovibrionales;f__Desulfovibrionaceae;g__Desulfovibrio;s__	0.077594	0.344468	0.053354	0.326881
bin.113	697	2553.31	1779655	3048	30.7668	78.71	1.342	Clostridiales	d__Bacteria;p__Firmicutes_A;c__Clostridia;o__Lachnospirales;f__Anaerotignaceae;g__An114;s__An114 sp002161055	0.060272	0.267572		
bin.114	238	9586.47	2281581	13584	51.0459	83.14	2.923	Lachnospiraceae	d__Bacteria;p__Firmicutes_A;c__Clostridia;o__Lachnospirales;f__Lachnospiraceae;g__Mediterraneibacter;s__Mediterraneibacter sp002314255				
bin.115	801	2438.83	1953501	2719	53.3424	69.08	5.477	Lachnospiraceae	d__Bacteria;p__Firmicutes_A;c__Clostridia;o__Lachnospirales;f__Lachnospiraceae;g__Mediterraneibacter;s__				
bin.116	243	7000.93	1701225	9157	26.8872	79.01	3.213	Clostridiales	d__Bacteria;p__Firmicutes_A;c__Clostridia;o__TANB77;f__CAG-508;g__CAG-354;s__	0.349285	1.550606	0.247897	1.518788
bin.117	399	5581.41	2226982	7246	49.6881	88	0.791	Clostridiales	d__Bacteria;p__Firmicutes_A;c__Clostridia;o__Lachnospirales;f__Lachnospiraceae;g__Blautia_A;s__				
bin.118	669	2824.72	1889741	3431	47.6763	72.55	0.994	Lachnospiraceae	d__Bacteria;p__Firmicutes_A;c__Clostridia;o__Lachnospirales;f__Lachnospiraceae;g__Mediterraneibacter;s__Mediterraneibacter sp002159505				
bin.119	149	23020.6	3430076	39672	49.5007	99.42	0.769	Bacteroidales	d__Bacteria;p__Bacteroidota;c__Bacteroidia;o__Bacteroidales;f__Tannerellaceae;g__Parabacteroides;s__Parabacteroides sp900552415	0.050258	0.223114	0.027242	0.166901
bin.12	289	6399.22	1849374	9137	61.4514	60.19	1.724	Bacteria	d__Bacteria;p__Firmicutes_A;c__Clostridia;o__Oscillospirales;f__Oscillospiraceae;g__UBA5446;s__UBA5446 sp900759385				
bin.120	41	45961.7	1884430	70466	46.6714	98.02	1.25	Proteobacteria	d__Bacteria;p__Proteobacteria;c__Gammaproteobacteria;o__Burkholderiales;f__Burkholderiaceae;g__CAG-521;s__CAG-521 sp000437635	0.316097	1.40327	0.259427	1.589425
bin.121	582	3357.77	1954222	4246	55.1766	91.32	4.496	Clostridiales	d__Bacteria;p__Firmicutes_A;c__Clostridia;o__Oscillospirales;f__Acutalibacteraceae;g__CAG-180;s__CAG-180 sp900545625	0.045087	0.200158	0.028417	0.174101
bin.122	439	3216.83	1412190	3519	55.7117	51.64	0	Bacteria	d__Bacteria;p__Firmicutes_B;c__Peptococcia;o__Peptococcales;f__Peptococcaceae;g__UMGS1590;s__UMGS1590 sp900552455				
bin.123	323	4938.65	1595184	6110	56.9665	85.48	0.735	Bacteria	d__Bacteria;p__Bacteroidota;c__Bacteroidia;o__Flavobacteriales;f__UBA1820;g__UBA1820;s__UBA1820 sp002314265	0.268522	1.192067	0.195187	1.195847
bin.124	478	4803.99	2296309	6780	63.0444	86.77	5.128	Bacteroidetes	d__Bacteria;p__Bacteroidota;c__Bacteroidia;o__Bacteroidales;f__Rikenellaceae;g__Alistipes;s__Alistipes sp900544265				
bin.125	268	5377.17	1441082	8142	49.6871	95.5	0.187	Bacteria	d__Bacteria;p__Elusimicrobiota;c__Elusimicrobia;o__Elusimicrobiales;f__Elusimicrobiaceae;g__CADBRU01;s__	0.662222	2.939847	0.532299	3.261222
bin.126	226	7235.83	1635298	10531	62.6303	93.75	0.832	Clostridia	d__Bacteria;p__Firmicutes_A;c__Clostridia_A;o__Christensenellales;f__Borkfalkiaceae;g__Borkfalkia;s__	0.053426	0.237178	0.023424	0.143513
bin.127	24	63634	1527215	104547	41.2696	91.47	0.447	Clostridiales	d__Bacteria;p__Firmicutes_A;c__Clostridia_A;o__Christensenellales;f__CAG-917;g__CAG-917;s__CAG-917 sp000437555	0.243129	1.079341	0.190302	1.165917
bin.128	539	4683.5	2524408	5457	63.295	87.53	2.822	Clostridia	d__Bacteria;p__Firmicutes_A;c__Clostridia_A;o__Christensenellales;f__CAG-74;g__JAAYOH01;s__	0.10557	0.468663		
bin.129	608	1793.49	1090444	1882	53.7482	57.92	1.662	Bacteria	d__Bacteria;p__Firmicutes;c__Bacilli;o__Erysipelotrichales;f__Erysipelotrichaceae;g__Merdibacter;s__				
bin.13	722	3204.38	2313559	4050	54.1427	78.6	4.563	Bacteroidales	d__Bacteria;p__Bacteroidota;c__Bacteroidia;o__Bacteroidales;f__Barnesiellaceae;g__Barnesiella;s__Barnesiella viscericola				
bin.130	908	1990.13	1807037	2158	47.6261	65.44	3.334	Bacteria	d__Bacteria;p__Verrucomicrobiota;c__Lentisphaeria;o__Victivallales;f__Victivallaceae;g__UMGS1518;s__	0.46759	2.075803		
bin.131	490	1988.71	974470	2102	29.3641	55.86	3.224	Clostridiales	d__Bacteria;p__Firmicutes_A;c__Clostridia;o__TANB77;f__CAG-508;g__CAG-273;s__CAG-273 sp900752335				
bin.132	989	1861.3	1840829	1937	35.0751	58.49	5.479	Bacteria	d__Bacteria;p__Cyanobacteria;c__Vampirovibrionia;o__Gastranaerophilales;f__Gastranaerophilaceae;g__CAG-484;s__	0.193161	0.857514	0.113923	0.697968
bin.133	780	2137.69	1667397	2363	52.7512	62.38	0	Bacteria	d__Bacteria;p__Firmicutes_A;c__Clostridia;o__Lachnospirales;f__Lachnospiraceae;g__Mediterraneibacter;s__				
bin.134	537	1882.05	1010662	1956	39.5158	57.53	1.088	Lactobacillales	d__Bacteria;p__Firmicutes;c__Bacilli;o__Lactobacillales;f__Streptococcaceae;g__Streptococcus;s__Streptococcus alactolyticus	0.144604	0.641952	0.12744	0.780785
bin.135	517	2604.96	1346762	3126	27.7124	70.87	3.033	Bacteria	d__Bacteria;p__Firmicutes;c__Bacilli;o__RF39;f__UBA660;g__CAG-460;s__				
bin.14	538	2950.34	1587283	3201	62.3949	61.45	1.724	Bacteria	d__Bacteria;p__Firmicutes_A;c__Clostridia;o__Oscillospirales;f__Acutalibacteraceae;g__Acutalibacter;s__Acutalibacter sp000435395				
bin.15	488	4747.32	2316692	6757	49.0136	90.29	2.275	Bacteroidales	d__Bacteria;p__Bacteroidota;c__Bacteroidia;o__Bacteroidales;f__Bacteroidaceae;g__UBA6398;s__UBA6398 sp900550635	0.1575	0.6992	0.101718	0.623194
bin.16	150	20419.8	3062967	36606	48.0784	96.82	2.237	Clostridiales	d__Bacteria;p__Firmicutes_A;c__Clostridia;o__Lachnospirales;f__Lachnospiraceae;g__Anaerobutyricum;s__	0.127842	0.567536		
bin.17	772	1950.24	1505588	1990	51.4821	62.69	2.684	Clostridiales	d__Bacteria;p__Firmicutes_A;c__Clostridia;o__Oscillospirales;f__Ruminococcaceae;g__UBA1448;s__				
bin.18	729	2943.73	2145980	3620	51.8807	84.8	3.313	Lachnospiraceae	d__Bacteria;p__Firmicutes_A;c__Clostridia;o__Lachnospirales;f__Lachnospiraceae;g__Mediterraneibacter;s__Mediterraneibacter sp900751785				
bin.19	603	3615.4	2180089	4688	62.7964	93.65	2.628	Clostridiales	d__Bacteria;p__Firmicutes_A;c__Clostridia;o__Oscillospirales;f__Acutalibacteraceae;g__Acutalibacter;s__	0.108165	0.480185	0.072802	0.446037
bin.2	66	27134.8	1790900	52600	34.0148	99.29	0.352	Campylobacterales	d__Bacteria;p__Campylobacterota;c__Campylobacteria;o__Campylobacterales;f__Helicobacteraceae;g__Helicobacter_D;s__Helicobacter_D pullorum	0.222004	0.985557	0.195096	1.195287
bin.20	174	11198.1	1948468	16589	48.6827	97.84	1.075	Bacteria	d__Bacteria;p__Proteobacteria;c__Alphaproteobacteria;o__RF32;f__CAG-239;g__CAG-495;s__CAG-495 sp000436375	0.565976	2.512577	0.400002	2.450685
bin.21	264	9462.38	2498067	16269	63.2563	91.89	0.705	Bacteria	d__Bacteria;p__Verrucomicrobiota;c__Kiritimatiellae;o__RFP12;f__UBA1067;g__W1P29-020;s__	0.461511	2.048819	0.437257	2.67893
bin.22	44	50625.1	2227506	82334	49.3406	66.66	0	Bacteroidales	d__Bacteria;p__Bacteroidota;c__Bacteroidia;o__Bacteroidales;f__Bacteroidaceae;g__Prevotella;s__Prevotella sp000433175	0.113461	0.503696	0.086614	0.530656
bin.23	522	3513.45	1834021	3904	54.6841	56.94	1.891	Lachnospiraceae	d__Bacteria;p__Firmicutes_A;c__Clostridia;o__Lachnospirales;f__Lachnospiraceae;g__Eisenbergiella;s__Eisenbergiella sp900544445				
bin.24	501	3893.32	1950553	5027	56.2315	86.47	1.23	Clostridiales	d__Bacteria;p__Firmicutes_A;c__Clostridia_A;o__Christensenellales;f__Borkfalkiaceae;g__Borkfalkia;s__	0.053306	0.236644	0.043878	0.268824
bin.25	1202	2235.46	2687024	2445	51.8629	71.66	1.344	Enterobacteriaceae	d__Bacteria;p__Proteobacteria;c__Gammaproteobacteria;o__Enterobacterales;f__Enterobacteriaceae;g__Escherichia;s__Escherichia flexneri	0.27422	1.217362	0.216525	1.326581
bin.26	171	13420.1	2294842	20693	53.1452	98.32	0	Clostridiales	d__Bacteria;p__Firmicutes_A;c__Clostridia;o__Oscillospirales;f__Acutalibacteraceae;g__;s__	0.103086	0.457638	0.057636	0.353116
bin.27	209	12581	2629433	21816	34.7063	98.96	0.919	Bacilli	d__Bacteria;p__Firmicutes;c__Bacilli;o__Bacillales;f__;g__;s__	0.202739	0.900033	0.140013	0.857814
bin.28	40	46644.9	1865798	105537	32.4696	89.74	0.854	Bacteria	d__Bacteria;p__Cyanobacteria;c__Vampirovibrionia;o__Gastranaerophilales;f__RUG14156;g__;s__	0.10908	0.484245	0.096848	0.593356
bin.29	350	2940.01	1029004	3214	29.406	54.88	2.105	Bacteria	d__Bacteria;p__Firmicutes;c__Bacilli;o__RF39;f__UBA660;g__CAG-460;s__				
bin.3	403	4285.58	1727088	5050	64.7205	75.52	1.342	Clostridiales	d__Bacteria;p__Firmicutes_A;c__Clostridia;o__Oscillospirales;f__Oscillospiraceae;g__UMGS1872;s__	0.243678	1.081775		
bin.30	439	3127.59	1373013	3226	36.3779	55.25	1.346	Bacilli	d__Bacteria;p__Firmicutes;c__Bacilli;o__Lactobacillales;f__Enterococcaceae;g__Enterococcus_E;s__Enterococcus_E cecorum	0.206738	0.917785	0.051119	0.31319
bin.31	105	32173.6	3378228	49353	44.2382	97.74	0.093	Bacteroidales	d__Bacteria;p__Bacteroidota;c__Bacteroidia;o__Bacteroidales;f__Bacteroidaceae;g__Phocaeicola;s__Phocaeicola plebeius_A				
bin.32	334	3763.41	1256978	4849	66.4639	53.57	1.785	Bacteria	d__Bacteria;p__Firmicutes_A;c__Clostridia;o__Oscillospirales;f__Ruminococcaceae;g__Faecalibacterium;s__				
bin.33	403	3786.94	1526137	5021	54.8798	87.18	0.418	Clostridiales	d__Bacteria;p__Firmicutes_A;c__Clostridia_A;o__Christensenellales;f__UBA3700;g__CABKMX01;s__	0.128426	0.570129	0.119326	0.73107
bin.34	59	44203.3	2607995	65485	47.6946	98.24	1.267	Lachnospiraceae	d__Bacteria;p__Firmicutes_A;c__Clostridia;o__Lachnospirales;f__Lachnospiraceae;g__Merdimonas;s__Merdimonas faecis	0.073129	0.324645	0.056732	0.347577
bin.35	39	46565.1	1816040	73782	62.1411	92.56	0.675	Bacteria	d__Bacteria;p__Verrucomicrobiota;c__Verrucomicrobiae;o__Opitutales;f__UBA953;g__W0P29-029;s__	0.522454	2.319365	0.472584	2.895368
bin.36	902	2165.87	1953618	2395	51.3306	68.4	3.02	Clostridiales	d__Bacteria;p__Firmicutes_A;c__Clostridia;o__Lachnospirales;f__Lachnospiraceae;g__Anaerobutyricum;s__				
bin.37	198	8866.38	1755543	13865	38.5565	99.18	5.452	Lactobacillales	d__Bacteria;p__Firmicutes;c__Bacilli;o__Lactobacillales;f__Lactobacillaceae;g__Limosilactobacillus;s__Limosilactobacillus reuteri_E	0.024465	0.108611	0.014125	0.086541
bin.38	788	2445.63	1927157	2805	51.198	75.68	3.996	Lachnospiraceae	d__Bacteria;p__Firmicutes_A;c__Clostridia;o__Lachnospirales;f__Lachnospiraceae;g__Massilistercora;s__				
bin.39	201	12348.2	2481979	17864	45.3388	98.24	2.514	Lachnospiraceae	d__Bacteria;p__Firmicutes_A;c__Clostridia;o__Lachnospirales;f__Lachnospiraceae;g__Sellimonas;s__Sellimonas sp002159995	0.085082	0.377712	0.03755	0.230057
bin.4	340	6350.97	2159331	8028	53.0697	91.81	2.767	Bacteroidales	d__Bacteria;p__Bacteroidota;c__Bacteroidia;o__Bacteroidales;f__UBA11471;g__UBA11471;s__UBA11471 sp900542765				
bin.40	434	4179.74	1814007	4928	47.8666	60.37	9.342	Lachnospiraceae	d__Bacteria;p__Firmicutes_A;c__Clostridia;o__Lachnospirales;f__Lachnospiraceae;g__Mediterraneibacter;s__				
bin.41	216	15371.9	3320332	30831	44.2203	98.46	0.499	Bacteroidales	d__Bacteria;p__Bacteroidota;c__Bacteroidia;o__Bacteroidales;f__Bacteroidaceae;g__Phocaeicola;s__Phocaeicola sp002161765	0.061659	0.273728	0.037148	0.227597
bin.42	270	12589.8	3399244	20909	35.92	99.36	0.632	Clostridiales	d__Bacteria;p__Firmicutes_A;c__Clostridia;o__Lachnospirales;f__Lachnospiraceae;g__CHKCI001;s__	0.102988	0.457202	0.068682	0.420791
bin.43	156	15695.9	2448562	28177	29.0374	92.76	0.943	Bacteria	d__Bacteria;p__Firmicutes;c__Bacilli;o__Erysipelotrichales;f__Erysipelatoclostridiaceae;g__Erysipelatoclostridium;s__Erysipelatoclostridium sp002160495				
bin.44	363	6348.69	2304574	12256	60.5	85.63	1.447	Bacteroidetes	d__Bacteria;p__Bacteroidota;c__Bacteroidia;o__Bacteroidales;f__Rikenellaceae;g__Alistipes;s__				
bin.45	46	44339.1	2039599	83265	49.8328	97.98	0	Clostridiales	d__Bacteria;p__Firmicutes_A;c__Clostridia;o__Oscillospirales;f__Acutalibacteraceae;g__CAG-180;s__	0.030248	0.13428	0.029412	0.180197
bin.46	429	5956.34	2555270	8192	45.0985	94.74	1.006	Clostridiales	d__Bacteria;p__Firmicutes_A;c__Clostridia;o__Lachnospirales;f__Anaerotignaceae;g__Anaerotignum;s__Anaerotignum lactatifermentans	0.087644	0.389083	0.150705	0.923321
bin.47	237	11711.8	2775697	17931	51.9655	89.91	2.531	Clostridiales	d__Bacteria;p__Firmicutes_A;c__Clostridia;o__Lachnospirales;f__Lachnospiraceae;g__Anaerosacchariphilus;s__Anaerosacchariphilus sp002160765	0.09281	0.412017		
bin.48	266	8897.94	2366851	12917	53.2849	97.8	1.265	Clostridiales	d__Bacteria;p__Firmicutes_A;c__Clostridia;o__Lachnospirales;f__Lachnospiraceae;g__OF09-33XD;s__	0.091131	0.404562		
bin.5	633	1708.75	1081641	1776	60.7312	70.08	6.993	Euryarchaeota	d__Archaea;p__Thermoplasmatota;c__Thermoplasmata;o__Methanomassiliicoccales;f__Methanomethylophilaceae;g__UBA71;s__UBA71 sp006954425	0.540044	2.397456	0.513316	3.144922
bin.50	546	2037.43	1112436	2227	50.1618	54.48	1.109	Clostridiales	d__Bacteria;p__Firmicutes_A;c__Clostridia_A;o__Christensenellales;f__CAG-917;g__UMGS1688;s__UMGS1688 sp900545885				
bin.51	378	6023.95	2277052	7973	47.2482	71.86	0	Bacteria	d__Bacteria;p__Firmicutes_A;c__Clostridia;o__Lachnospirales;f__Lachnospiraceae;g__Mediterraneibacter;s__				
bin.52	126	15137.3	1907302	25899	40.2826	98.91	0.425	Lactobacillales	d__Bacteria;p__Firmicutes;c__Bacilli;o__Lactobacillales;f__Lactobacillaceae;g__Limosilactobacillus;s__Limosilactobacillus vaginalis	0.0334	0.148276	0.024058	0.147394
bin.53	97	15821.4	1534678	34654	50.6277	97.05	1.307	Euryarchaeota	d__Archaea;p__Halobacteriota;c__Methanomicrobia;o__Methanomicrobiales;f__Methanocorpusculaceae;g__Methanocorpusculum;s__	0.47344	2.101773	0.412921	2.529835
bin.54	177	14843.6	2627310	23216	28.8046	98.79	0.134	Bacteria	d__Bacteria;p__Firmicutes;c__Bacilli;o__Erysipelotrichales;f__Erysipelatoclostridiaceae;g__Erysipelatoclostridium;s__Erysipelatoclostridium spiroforme	0.376398	1.670967	0.199689	1.22343
bin.55	97	22332.8	2166278	34786	61.9917	96.74	1.949	Bacteroidetes	d__Bacteria;p__Bacteroidota;c__Bacteroidia;o__Bacteroidales;f__Rikenellaceae;g__Alistipes;s__				
bin.56	390	3671.6	1431923	4308	62.819	57.55	0	Bacteria	d__Bacteria;p__Firmicutes_A;c__Clostridia;o__Oscillospirales;f__Oscillospiraceae;g__UBA5446;s__UBA5446 sp900543085				
bin.57	360	8012.77	2884598	10279	52.8104	86.59	1.292	Clostridiales	d__Bacteria;p__Firmicutes_A;c__Clostridia;o__Lachnospirales;f__Lachnospiraceae;g__Enterocloster;s__				
bin.58	350	6155.84	2154544	7738	66.8069	90.73	3.379	Clostridiales	d__Bacteria;p__Firmicutes_A;c__Clostridia_A;o__Christensenellales;f__CAG-74;g__OEMS01;s__				
bin.59	98	18251.4	1788639	34663	45.6896	90.72	0	Clostridia	d__Bacteria;p__Firmicutes_A;c__Clostridia_A;o__Christensenellales;f__CAG-314;g__UMGS929;s__UMGS929 sp900546875	0.276629	1.228057	0.22167	1.358102
bin.6	617	3153.7	1945833	4081	49.1662	78.44	3.947	Lachnospiraceae	d__Bacteria;p__Firmicutes_A;c__Clostridia;o__Lachnospirales;f__Lachnospiraceae;g__Mediterraneibacter;s__				
bin.60	277	5981.11	1656768	7503	51.0118	58.02	5.172	Bacteria	d__Bacteria;p__Firmicutes_A;c__Clostridia;o__Lachnospirales;f__Lachnospiraceae;g__UBA7182;s__UBA7182 sp002160135				
bin.61	567	3294.99	1868262	4186	37.8007	83.01	2.051	Bacteria	d__Bacteria;p__Cyanobacteria;c__Vampirovibrionia;o__Gastranaerophilales;f__;g__;s__	0.185205	0.822193		
bin.62	226	8547.35	1931700	10348	49.7529	64.96	1.724	Bacteria	d__Bacteria;p__Firmicutes_A;c__Clostridia;o__Lachnospirales;f__Lachnospiraceae;g__Mediterraneibacter;s__				
bin.63	613	4653.02	2852304	5993	59.4402	89.85	3.681	Clostridia	d__Bacteria;p__Firmicutes_A;c__Clostridia_A;o__Christensenellales;f__CAG-74;g__HGM11588;s__HGM11588 sp900754755	0.137542	0.610599	0.226296	1.386443
bin.64	440	7039.62	3097431	9943	64.3504	98.52	2.684	Deltaproteobacteria	d__Bacteria;p__Desulfobacterota;c__Desulfovibrionia;o__Desulfovibrionales;f__Desulfovibrionaceae;g__Desulfovibrio;s__	0.082487	0.366188	0.030648	0.18777
bin.65	113	12348.4	1395372	20889	29.046	94.94	1.123	Bacteria	d__Bacteria;p__Firmicutes;c__Bacilli;o__RF39;f__UBA660;g__RUG591;s__	0.188655	0.837509	0.132087	0.809255
bin.67	554	2169.57	1201942	2429	51.5664	69.87	0.244	Clostridiales	d__Bacteria;p__Firmicutes_A;c__Clostridia;o__Oscillospirales;f__Ruminococcaceae;g__UBA1409;s__UBA1409 sp002305045	0.260747	1.157553		
bin.68	1002	2150.9	2155201	2337	60.9795	64.12	8.103	Bacteria	d__Bacteria;p__Firmicutes_A;c__Clostridia;o__Oscillospirales;f__Butyricicoccaceae;g__Agathobaculum;s__				
bin.69	30	59479	1784371	79561	60.4402	98.79	0	Bacteroidetes	d__Bacteria;p__Bacteroidota;c__Bacteroidia;o__Bacteroidales;f__Rikenellaceae;g__Tidjanibacter;s__	0.160628	0.713087	0.083361	0.510728
bin.7	740	4242.33	3139327	6779	45.1578	92.22	1.613	Clostridiales	d__Bacteria;p__Firmicutes_A;c__Clostridia;o__Lachnospirales;f__Lachnospiraceae;g__Blautia;s__	0.062916	0.279308	0.051748	0.317043
bin.70	797	1972.89	1572391	2134	62.359	72.14	3.597	Clostridiales	d__Bacteria;p__Firmicutes_A;c__Clostridia;o__Oscillospirales;f__Acutalibacteraceae;g__UBA1417;s__UBA1417 sp900552925	0.075603	0.335631	0.063013	0.386063
bin.71	531	4140.37	2198535	5983	64.1498	85.35	3.968	Clostridiales	d__Bacteria;p__Firmicutes_A;c__Clostridia;o__Oscillospirales;f__Ruminococcaceae;g__Faecalibacterium;s__				
bin.72	95	25692.7	2440810	43016	50.9037	98.43	0	Clostridiales	d__Bacteria;p__Firmicutes_A;c__Clostridia;o__Oscillospirales;f__Acutalibacteraceae;g__;s__	0.146835	0.651855	0.096428	0.590784
bin.73	132	26522.2	3500925	59463	51.3093	97.67	0.557	Bacteroidales	d__Bacteria;p__Bacteroidota;c__Bacteroidia;o__Bacteroidales;f__Bacteroidaceae;g__Bacteroides;s__	0.054563	0.242224	0.039032	0.239134
bin.74	310	8577.34	2658976	11105	50.7662	87.65	2.056	Clostridiales	d__Bacteria;p__Firmicutes_A;c__Clostridia;o__Lachnospirales;f__Lachnospiraceae;g__UMGS1370;s__				
bin.75	459	5562.58	2553225	8452	63.156	80.04	1.075	Clostridia	d__Bacteria;p__Firmicutes_A;c__Clostridia_A;o__Christensenellales;f__CAG-74;g__Firm-11;s__				
bin.76	318	8062.72	2563945	13598	58.3348	97.44	0.632	Clostridiales	d__Bacteria;p__Firmicutes_A;c__Clostridia;o__Lachnospirales;f__Lachnospiraceae;g__Lachnoclostridium_A;s__	0.097821	0.434265	0.059071	0.361907
bin.77	573	3226.87	1848995	3515	58.5537	67.39	0.862	Bacteria	d__Bacteria;p__Firmicutes_A;c__Clostridia;o__Oscillospirales;f__Oscillospiraceae;g__UMGS1872;s__				
bin.78	410	4818.12	1975429	6106	66.2156	79.94	1.344	Actinobacteria	d__Bacteria;p__Actinobacteriota;c__Coriobacteriia;o__Coriobacteriales;f__Eggerthellaceae;g__UMGS1293;s__UMGS1293 sp900754495	0.386554	1.716054	0.362135	2.218684
bin.79	614	6359.57	3904774	8533	45.1742	90.98	0.128	Bacteroidales	d__Bacteria;p__Bacteroidota;c__Bacteroidia;o__Bacteroidales;f__Tannerellaceae;g__Parabacteroides;s__Parabacteroides johnsonii	0.045156	0.200463	0.038053	0.23314
bin.8	415	4864.21	2018649	5899	57.4566	92.99	1.342	Clostridiales	d__Bacteria;p__Firmicutes_A;c__Clostridia;o__Oscillospirales;f__Ruminococcaceae;g__Negativibacillus;s__Negativibacillus sp900547455	0.154335	0.685149	0.118291	0.724729
bin.80	13	53503.8	695549	170417	55.6173	61.17	0.854	Bacteria	d__Bacteria;p__Patescibacteria;c__Saccharimonadia;o__Saccharimonadales;f__Saccharimonadaceae;g__UBA2866;s__	0.738053	3.276487	0.578682	3.545396
bin.81	22	75989.9	1671777	112474	30.7192	99.43	0.186	Campylobacter	d__Bacteria;p__Campylobacterota;c__Campylobacteria;o__Campylobacterales;f__Campylobacteraceae;g__Campylobacter_D;s__Campylobacter_D coli	0.193162	0.857517	0.134737	0.825492
bin.82	734	2402.77	1763635	2698	53.1228	79.49	1.745	Clostridiales	d__Bacteria;p__Firmicutes_A;c__Clostridia;o__Lachnospirales;f__Lachnospiraceae;g__;s__				
bin.83	261	8265.84	2157385	13311	44.0417	94.63	2.237	Clostridiales	d__Bacteria;p__Firmicutes_A;c__Clostridia;o__Lachnospirales;f__Lachnospiraceae;g__Anaerostipes;s__	0.116185	0.51579	0.099656	0.61056
bin.84	275	5570.64	1531925	8843	54.8959	89.26	0.803	Clostridiales	d__Bacteria;p__Firmicutes_A;c__Clostridia_A;o__Christensenellales;f__UBA3700;g__CABKMX01;s__	0.148369	0.658663	0.135438	0.829787
bin.85	536	2495.1	1337376	2477	63.602	52.83	2.432	Clostridiales	d__Bacteria;p__Firmicutes_A;c__Clostridia;o__Oscillospirales;f__Oscillospiraceae;g__Lawsonibacter;s__Lawsonibacter sp900545895				
bin.87	193	15486.4	2988879	26351	48.7125	97.5	0.316	Clostridiales	d__Bacteria;p__Firmicutes_A;c__Clostridia;o__Lachnospirales;f__Lachnospiraceae;g__UMGS1370;s__				
bin.88	505	2796.74	1412352	3265	48.8472	73.35	1.577	Clostridia	d__Bacteria;p__Firmicutes_A;c__Clostridia_A;o__Christensenellales;f__DTU072;g__;s__	0.256987	1.140859	0.195347	1.19683
bin.89	448	6444.27	2887033	11740	63.0231	90.76	2.276	Clostridia	d__Bacteria;p__Firmicutes_A;c__Clostridia_A;o__Christensenellales;f__CAG-74;g__Firm-11;s__Firm-11 sp900553905				
bin.9	1034	2259.32	2336140	2549	52.7538	76.29	1.371	Clostridiales	d__Bacteria;p__Firmicutes_A;c__Clostridia;o__Lachnospirales;f__Lachnospiraceae;g__;s__	0.050089	0.222363		
bin.90	126	18908	2382411	36128	50.0828	98.39	0.68	Clostridiales	d__Bacteria;p__Firmicutes_A;c__Clostridia;o__Oscillospirales;f__Ruminococcaceae;g__Ruthenibacterium;s__	0.091256	0.405121	0.049719	0.304611
bin.91	310	4937.12	1530506	6796	29.5841	85.39	2.407	Bacteria	d__Bacteria;p__Firmicutes;c__Bacilli;o__RF39;f__UBA660;g__CAG-877;s__	0.16332	0.725037	0.129295	0.79215
bin.93	382	4659.28	1779844	6046	59.7334	85.36	3.712	Clostridiales	d__Bacteria;p__Firmicutes_A;c__Clostridia_A;o__Christensenellales;f__Borkfalkiaceae;g__Borkfalkia;s__	0.053725	0.238506	0.046506	0.284928
bin.94	71	36527.7	2593467	56842	49.2756	98.73	0.471	Bacteroidales	d__Bacteria;p__Bacteroidota;c__Bacteroidia;o__Bacteroidales;f__Barnesiellaceae;g__Barnesiella;s__	0.077524	0.344156	0.06035	0.369744
bin.95	297	6098.25	1811180	10809	34.1268	98.19	2.928	Lactobacillus	d__Bacteria;p__Firmicutes;c__Bacilli;o__Lactobacillales;f__Lactobacillaceae;g__Lactobacillus;s__Lactobacillus johnsonii	0.069605	0.309004	0.05282	0.323612
bin.96	547	3499.06	1913987	4338	46.6676	92.06	1.006	Bacteroidales	d__Bacteria;p__Bacteroidota;c__Bacteroidia;o__Bacteroidales;f__UBA11471;g__;s__	0.10793	0.47914	0.094377	0.578219
bin.97	74	31413.2	2324577	55134	51.048	96.87	0.48	Bacteroidetes	d__Bacteria;p__Bacteroidota;c__Bacteroidia;o__Bacteroidales;f__Rikenellaceae;g__Alistipes_A;s__Alistipes_A sp900546005	0.113226	0.502652	0.066954	0.410204
bin.98	278	6907.81	1920370	9328	50.2956	88.88	0.632	Clostridiales	d__Bacteria;p__Firmicutes_A;c__Clostridia;o__Lachnospirales;f__Lachnospiraceae;g__Ruminococcus_G;s__	0.051637	0.229237	0.036827	0.225626
bin.99	269	8093.1	2177043	12818	61.5381	84.01	1.875	Bacteroidetes	d__Bacteria;p__Bacteroidota;c__Bacteroidia;o__Bacteroidales;f__Rikenellaceae;g__Alistipes;s__Alistipes sp002161445				

**Fig. 3:**
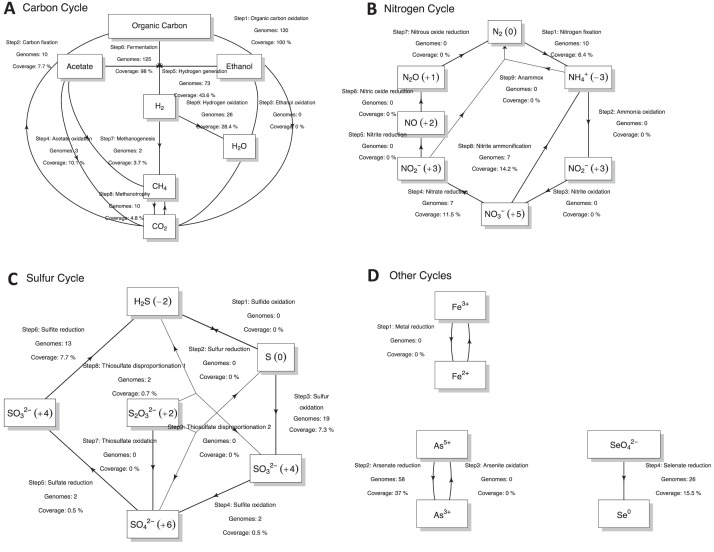
Major biogeochemical cycles recovered for 130 MAGs: (A) Carbon Cycle, (B) Nitrogen Cycle, (C) Sulfur Cycle, and (D) Other Cycles. Each arrow represents a single transformation. Besides each arrow is given the number of MAGs that can conduct these reactions and the metagenomic coverages (as a percentage of total MAGs).

**Fig. 4:**
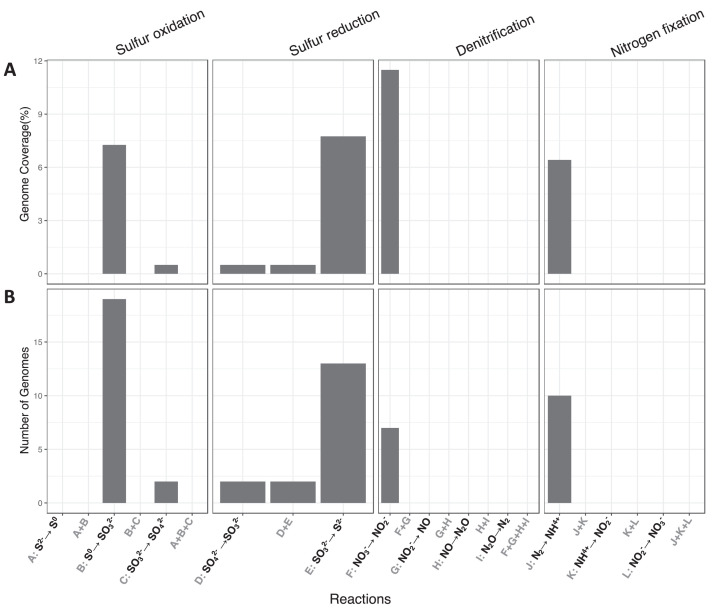
Schematic figure of sequential metabolic transformations of inorganic compounds recovered for 130 MAGs. X-axes describe individual sequential transformation indicated by letters. The two panels describe the (A) number of MAGS and (B) genomes coverages (proportion of total MAGs) that are involved in a certain sequential process.

**Fig. 5:**
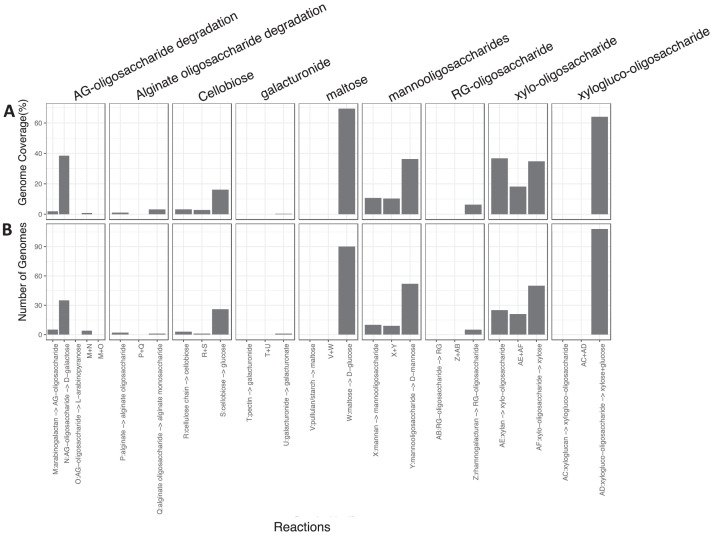
Schematic figure of sequential metabolic transformations of organic compounds recovered for 130 MAGs. X-axes describe individual sequential transformation indicated by letters. The two panels describe the (A) number of MAGS and (B) genomes coverages (proportion of total MAGs) that are involved in a certain sequential process.

To obtain metabolic functions, nutrient cycling diagrams (Carbon, Sulfur, including taxonomy using GTDB-TK [8], we used the METABOLIC pipeline [9]. METABOLIC allowed for the recovery of annotated proteins using KEGG [10], TIGRfam [11], Pfam [12], custom hidden Markov model (HMM) databases [13], dbCAN2 [14], and MEROPS [15]. Fig. 3, Fig. 4, Fig. 5 provide the genomes and coverages for different biogeochemical cycles, including the degradation of inorganic and organic compounds.

To infer the phylogeny of the MAGs, we have used the GToTree [16]. The software provides several Single Copy Genes (SCGs) sets depending on the resolution of the domains and taxonomic rank of interest. We have used two SCG sets, a 25-gene Bacteria and Archaea SCG set (recovered phylogeny for 81 MAGs) and a 16 genes SCG set (recovered phylogeny for 70 MAGs) by [17] that covers all major domains of life. To see which MAGs are novel, we have used the Genome Tree Toolkit (https://github.com/donovan-h-parks/GenomeTreeTk) by checking the phylogenetic gain for each MAG against the rest of the tree, with higher values potentially identifying novel species. We calculated these for each MAG in the trees recovered for both the 25 genes Bacteria and Archaea SCGs, and the 16 genes SCGs from [17], respectively.

## 4 Data Accessibility

The FASTA files, tables, annotations, and visualisations are provided at Figshare: http://dx.doi.org/10.6084/m9.figshare.24901878.

## Ethics Statement

This study was approved by the Ethics Review Board (ERB) at COMSATS University Islamabad (ERB No. CUI/Bio/ERB-4-21/17/).

## CRediT authorship contribution statement

**Aqsa Ameer:** Conceptualization, Methodology, Validation, Formal analysis, Investigation, Data curation, Writing – original draft, Visualization. **Farrukh Saleem:** Conceptualization, Methodology, Validation, Formal analysis, Investigation, Data curation, Writing – original draft, Visualization. **Ciara Keating:** Funding acquisition, Resources, Writing – review & editing, Data curation. **Ozan Gundogdu:** Resources, Writing – review & editing, Data curation. **Umer Zeeshan Ijaz:** Software, Validation, Formal analysis, Resources, Writing – original draft, Supervision, Project administration, Funding acquisition. **Sundus Javed:** Conceptualization, Methodology, Resources, Writing – review & editing, Supervision, Funding acquisition.

## Data Availability

Dataset of 130 metagenome-assembled genomes of healthy and diseased broiler chicken caeca from Pakistan (Original data) (Figshare).
